# Supplementary material for: Thermally trainable dual network hydrogels
Source: Nat Commun. 2023 Jun 22;14:3717. doi: 10.1038/s41467-023-39446-w (PMC10287690; doi:10.1038/s41467-023-39446-w)
Supplement: Supplementary file 1 — Supplementray Information [file 41467_2023_39446_MOESM1_ESM.pdf]

Supplementary Information

Thermally Trainable Dual Network Hydrogels

Shanming Hu[1], Yuhuang Fang[1], Chen Liang[1], Matti Turunen[1], Olli Ikkala[1]*, and Hang Zhang[1]*

[1] Department of Applied Physics, Aalto University, P.O. Box 15100, Espoo FI 02150, Finland

Table of Contents

1. Supplementary text 3
2. Supplementary figures 5
3. Reference 27

1. Supplementary text

Fitting of swelling/shrinking kinetics. The fitting of the swelling and shrinking kinetics was carried out in Origin according to Equation S1.

$$y = y_0 + A_1 e^{(-\frac{x-x_0}{t_1})} \qquad \text{(S1)}$$

where y is the swelling ratio, y_0, A_1, x_0, and t_1 are constants to be fitted, and x is the time in seconds. t_1 represents the time constant of the exponential decay. The fitted t_1 are summarized in Supplementary Table 1 for both positive and negative training hydrogels.

Supplementary Table 1 Fitted t_1 for swelling/shrinking kinetics of hydrogels

	t_1 – shrinking (min)		t_1 – swelling (min)	
	25 → 40 °C	25 → 70 °C	40 → 25°C	70 → 25 °C
Positive training hydrogel	1.22 ± 0.08	0.44 ± 0.03	4.53 ± 1.16	11.87 ± 3.21
Negative training hydrogel	1.37 ± 1.01	0.11 ± 0.13	7.58 ± 0.05	7.29 ± 0.91

Fitting of training kinetics for positive training hydrogels. The fitting of the training kinetics was carried out in Origin according to Equation S2.

$$y = y_0 + A_2 e^{(-\frac{x-x_0}{t_2})} \qquad \text{(S2)}$$

where y is the swelling ratio, y_0, A_2, x_0, and t_2 are constants to be fitted, and x is the number of cycles. t_2 represents the time constant of the exponential decay. The fitted t_2 are summarized in Supplementary Table 2. The fitted curves are shown in Supplementary Figure 14.

Supplementary Table 2 Fitted t_2 for training kinetics of positive training hydrogels

agarose concentration	1 wt% - 50°C	2 wt%		3 wt% - 50°C
		50°C	70°C	
t_2	1.95 ± 0.49	2.06 ± 0.58	1.34 ± 0.10	0.73 ± 0.11

Calculation of degree of substitution. Appearance of proton signals from acryloyl groups (-CO-CH=CH2) in ^{1}H NMR spectrum between 5.9 ppm and 6.4 ppm indicated the partial acrylation of agarose[1].The degree of substitution was calculated from the integral ratio of the signals at 5.9–6.4 ppm compared to that of the signals from protons at position 1 in 3,6 anhydro-

α-L-galactopyranose unit of agarose (red highlighted in Supplementary Fig. 15) at around 5.1 ppm, which were used as internal standard[2].

Supplementary Table 3 Feed ratio and degree of substitution calculated from NMR spectra.

Feed ratio	10 %	15 %	30 %	50 %
Degree of substitution	0.7 %	1.1 %	2.3 %	2.8 %

Fitting of training kinetics for negative training hydrogels. The fitting of the training kinetics was carried out in Origin according to Equation S2. The fitted t_2 are summarized in Supplementary Table 4, and the fitted curves are shown in Supplementary Figure 19.

Supplementary Table 4 Fitted t_2 for training kinetics of negative training hydrogels

ac-agarose concentration	1 wt% - 50°C	2 wt%		3 wt% - 50°C
		50°C	70°C	
t_2	0.99 ± 0.09	1.27 ± 0.20	1.59 ± 0.30	0.78 ± 0.07

2. Supplementary figures

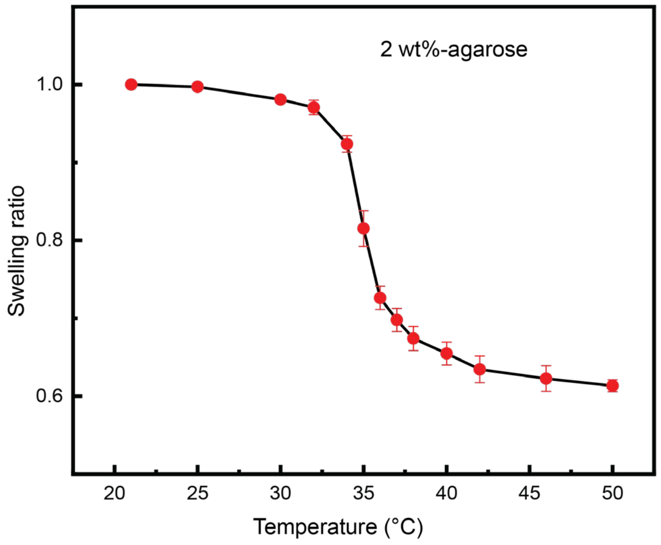

Supplementary Figure 1. Temperature-dependent swelling ratio of positive training hydrogel. The positive training hydrogel consists of 2 wt% agarose and 10 wt% PNIPAm, showing a LCST at around 35°C. Hydrogels were equilibrated at each temperature for 30 min. Error bars represent standard deviations from 4 measurements.

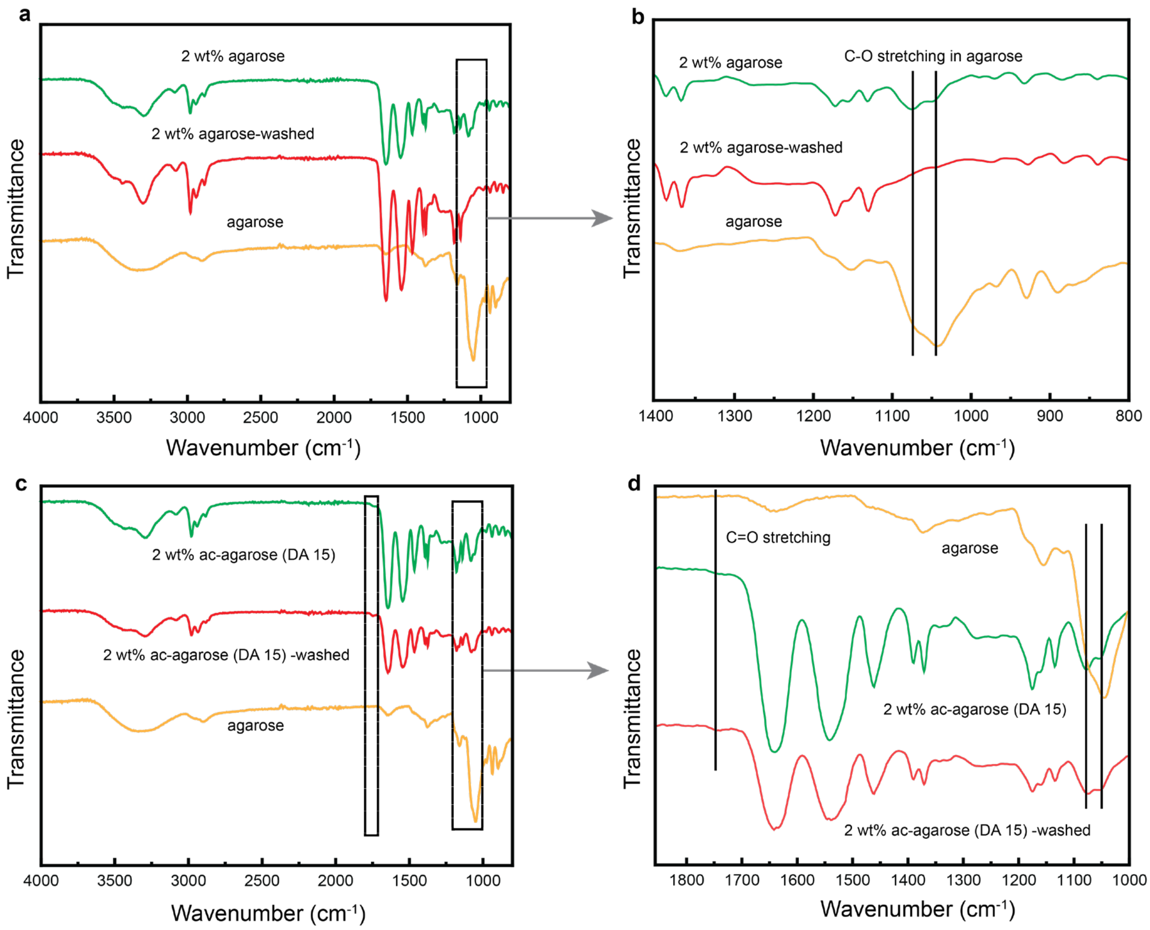

Supplementary Figure 2. FTIR characterization of thermally trainable hydrogels. a The FTIR spectra of pure agarose (yellow line), positive training hydrogels containing 2 wt% agarose before (green line) and after (red line) training. **b** Magnification of the area between 800 and 1400 cm^{-1} in (**a**). The bands between 1000 and 1100 cm^{-1} (C-O stretching, highlighted by black lines) disappeared in the hydrogels after training, confirming the removal of the agarose. **c** The FTIR spectra of pure agarose (yellow line) and negative training hydrogels before (green line) and after (red line) training. **d** Magnification of the area between 800 and 1400 cm^{-1} as in (**c**). The bands between 1000 and 1100 cm^{-1} (C-O stretching, highlighted by back lines) still existed in the after training hydrogels, confirming the preservation of ac-agarose in the hydrogels. In addition, the absorption peak at 1720 cm^{-1} can be attributed to C=O stretching of the acrylate groups.

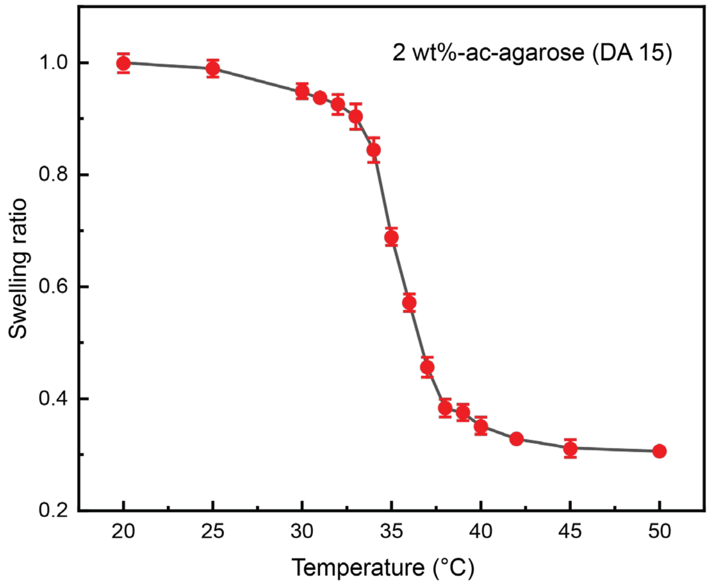

Supplementary Figure 3. Temperature-dependent swelling ratio of negative training hydrogel. The negative training hydrogel consists of 2 wt% ac-agarose (DA 15) and 10 wt% PNIPAm, showing a LCST at around 35°C. Hydrogels were equilibrated at each temperature for 30 min. Error bars represent standard deviations from 4 measurements.

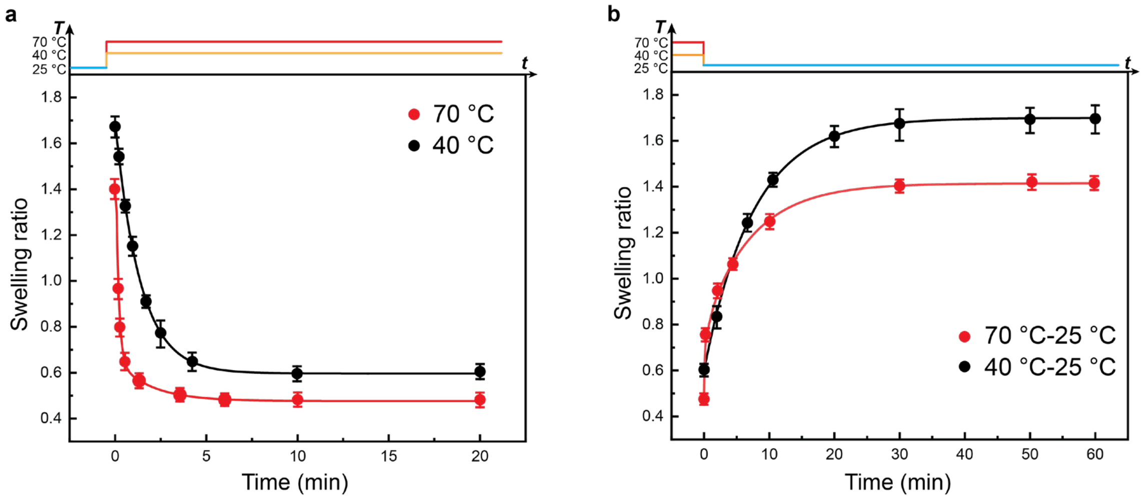

Supplementary Figure 4. Shrinking and swelling kinetics of negative training hydrogels. **a** Heating from RT to 40 or 70°C. Note that the sample heated to 70°C was pre-trained for 3 cycles before the measurement. The sample heated to 40 °C was the pristine hydrogel. **b** Cooling from 40 or 70°C to RT. Note that the sample cooled from 70°C to RT was pre-trained for 3 cycles before the measurement. The sample cooled from 40 °C to RT was the pristine hydrogel. Hydrogels were prepared with 2 wt% ac-agarose (DA 15) and 10 wt% PNIPAm. Error bars represent standard deviations from 4 measurements.

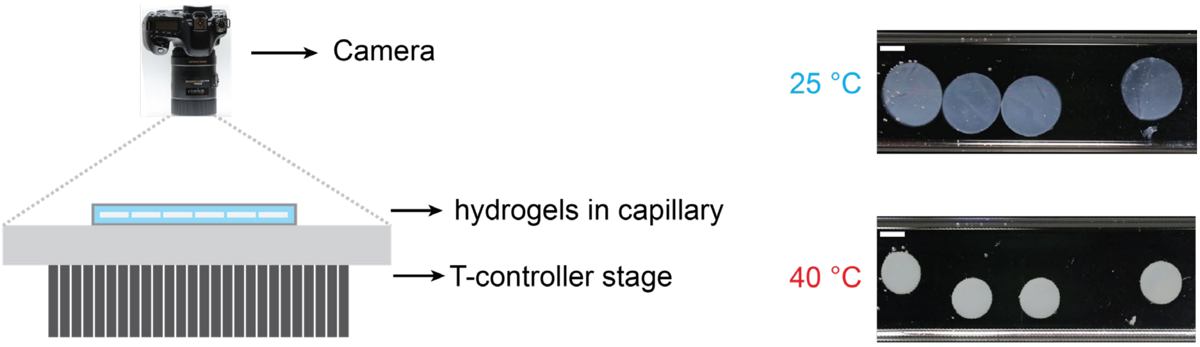

Supplementary Figure 5. Experimental setup for swelling/shrinking characterization of hydrogels. Representative photos of the hydrogel disks are included. Scale bar = 3 mm.

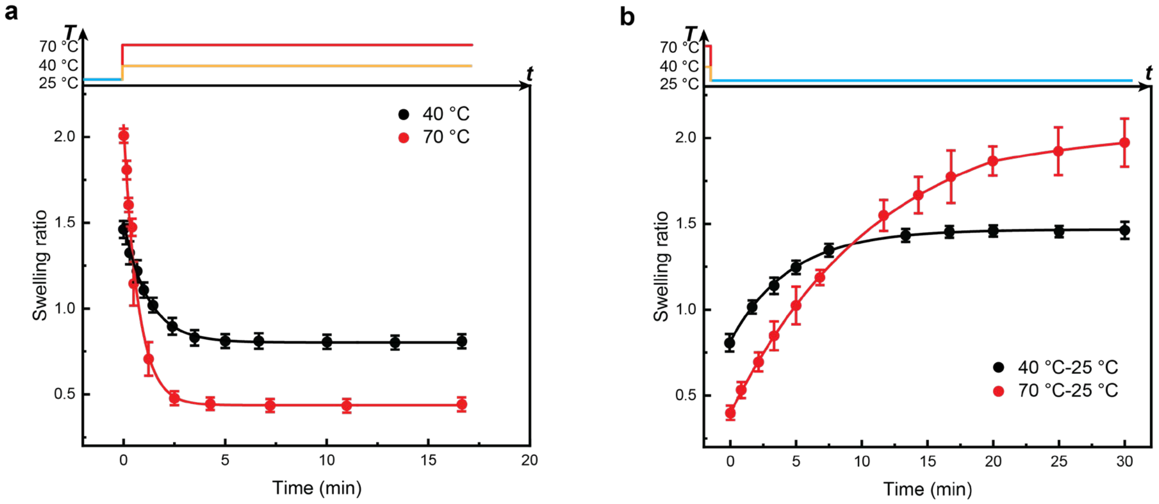

Supplementary Figure 6. The swelling and shrinking kinetics of positive training hydrogels. a Heating from RT to 40 or 70°C. Note that the sample heated to 70°C was pre-trained for 3 cycles before the measurement. The sample heated to 40 °C was the pristine hydrogel. **b** Cooling from 40 or 70°C to RT. Note that the sample cooled from 70°C to RT was pre-trained for 3 cycles before the measurement. The sample cooled from 40 °C was the pristine hydrogel. DN agarose/PNIPAm hydrogels containing 2 wt% agarose were used. Error bars represent standard deviations from 4 measurements.

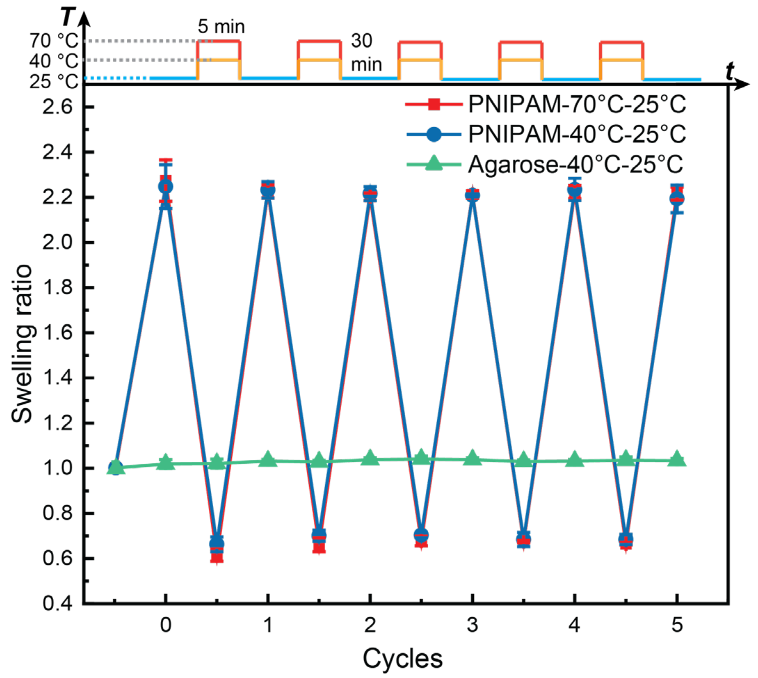

Supplementary Figure 7. Control experiment of training of pure PNIPAM and agarose hydrogels. Training cycles were repeated for 5 times, each consisting of 5 min at 40°C or 70°C and 30 min at 25 °C. Error bars are standard deviation from 4 measurements.

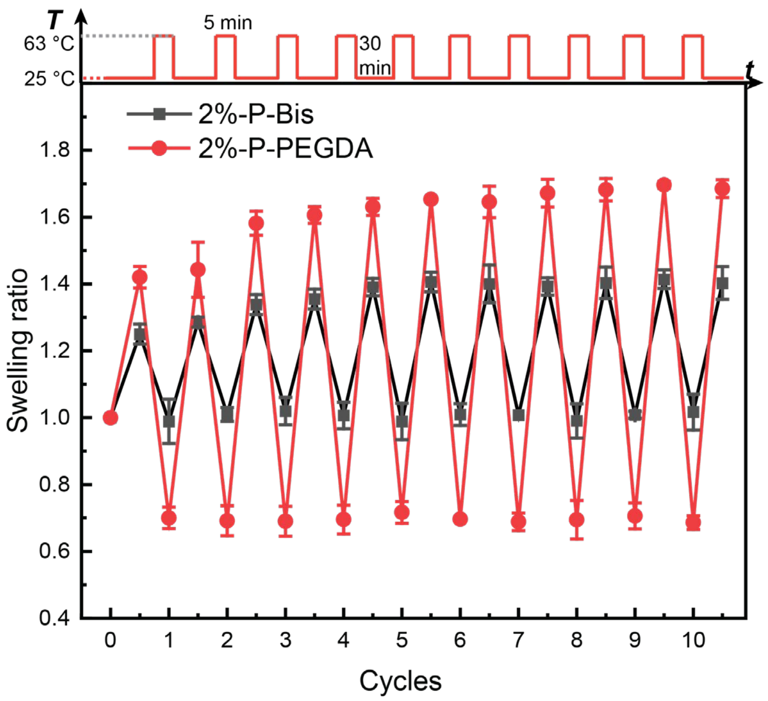

Supplementary Figure 8. Crosslinker effect on the swelling ratio. Positive training hydrogels were prepared with different crosslinkers: poly (ethylene glycol) diacrylate (PEGDA, M_n = 10 000) or *N, N′*-methylenebisacrylamide (BIS). Error bars are standard deviations from 4 measurements.

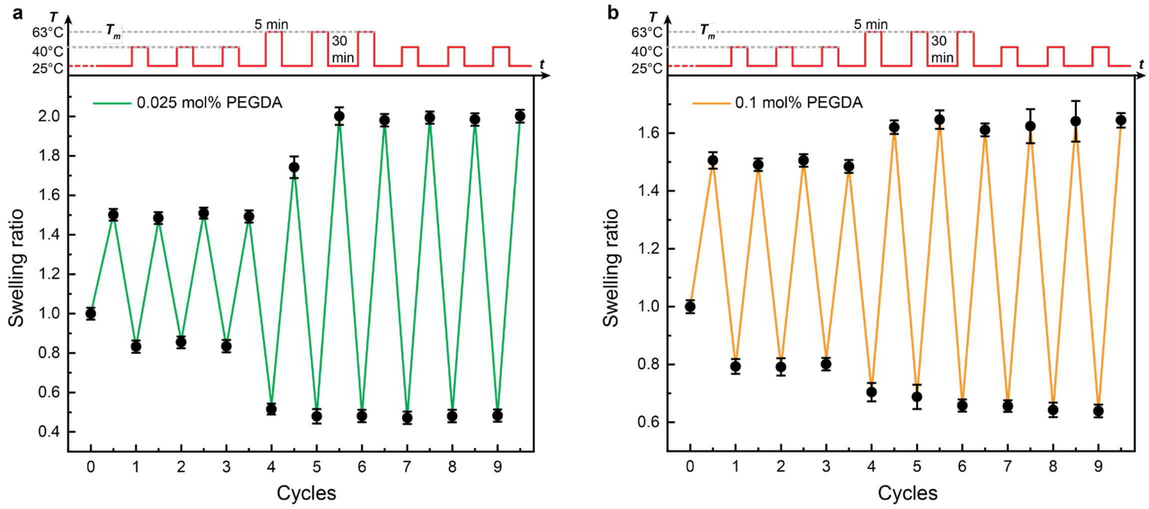

Supplementary Figure 9. Effect of crosslinking density on the swelling ratio in positive training hydrogels. The hydrogels containing 2 wt% agarose were prepared with different crosslinking density: 0.025 mol% (**a**) or 0.1 mol% (**b**) PEGDA relative to NIPAm. Error bars are standard deviations from 4 measurements.

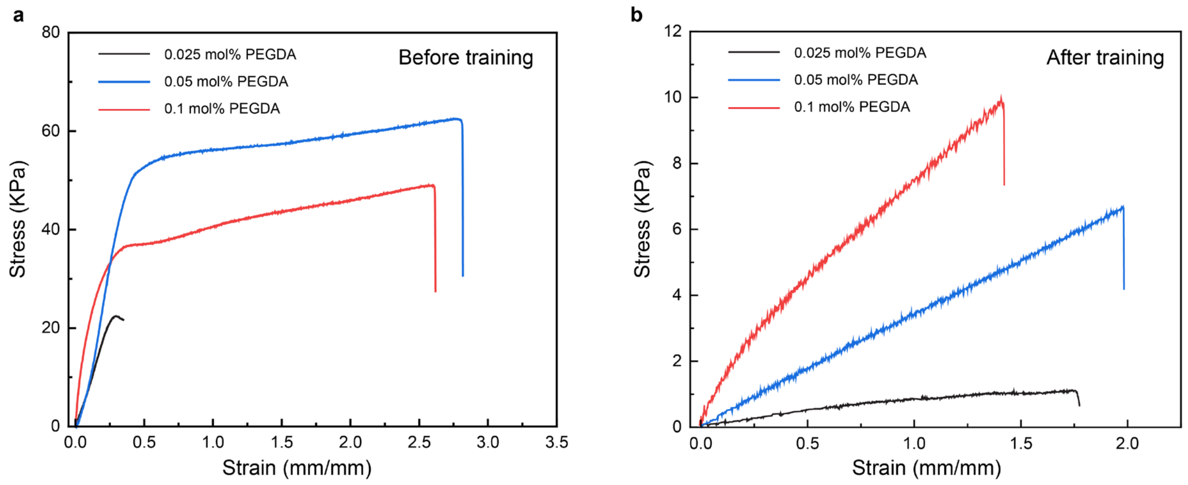

Supplementary Figure 10. Effect of crosslinking density on the mechanical properties in positive training hydrogels. a Tensile tests of pristine hydrogels before training. **b** Tensile tests of hydrogels after training for 3 cycles at 70°C. The hydrogels containing 2 wt% agarose were prepared with different crosslinking density: 0.025 mol%, 0.05 mol% or 0.1 mol% PEGDA relative to NIPAm (10 wt%).

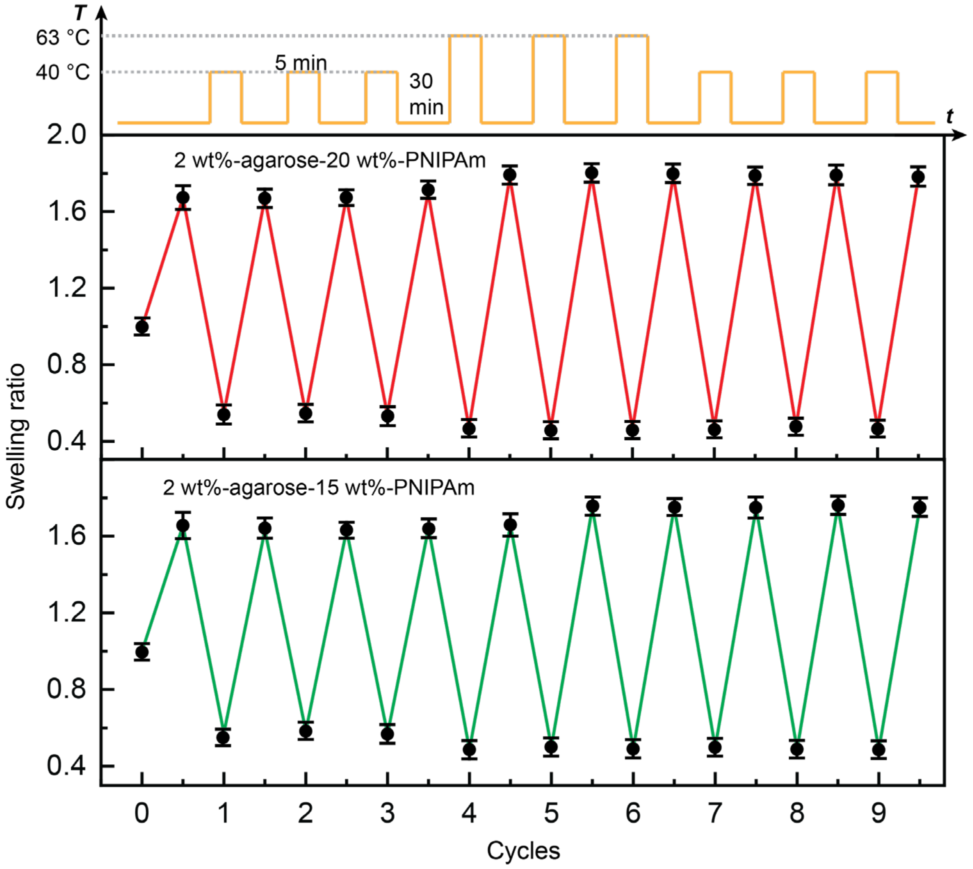

Supplementary Figure 11. Training of hydrogels containing higher PNIPAm content. The hydrogel consists of 2 wt% agarose, 15 wt% or 20 wt% PNIPAm, and 0.05 mol % PEGDA relative to NIPAm.

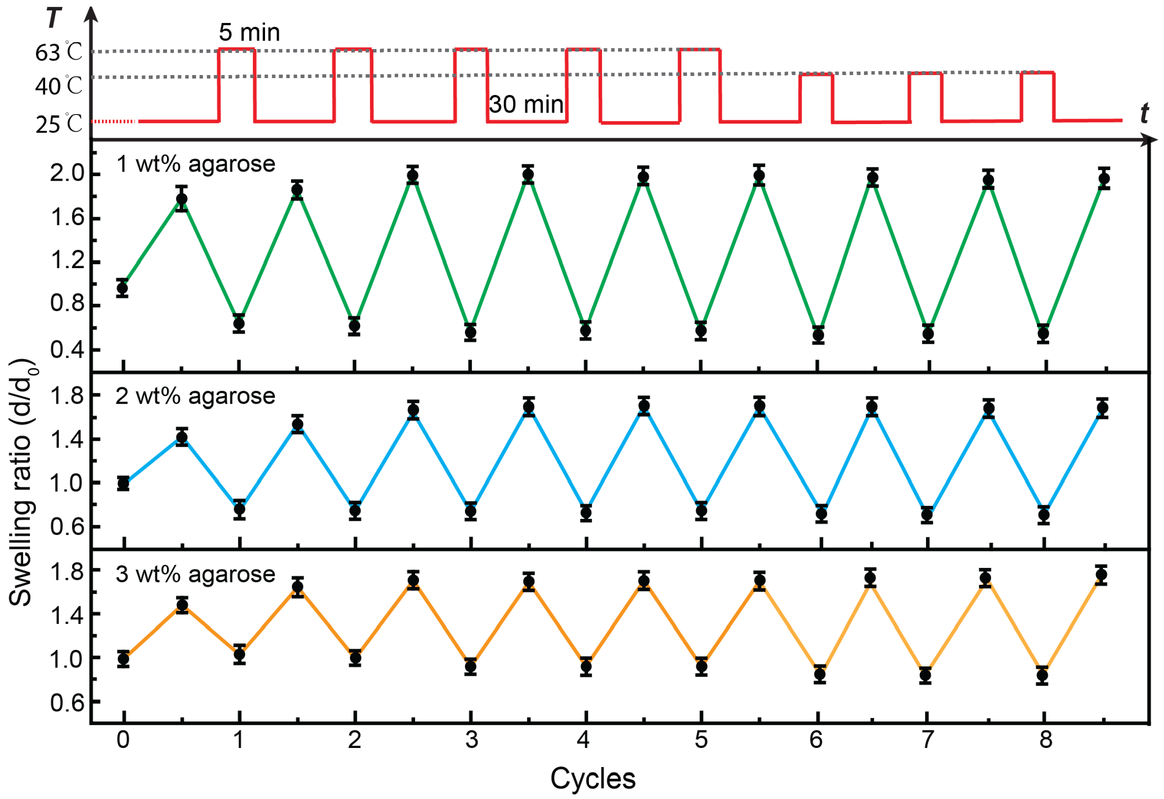

Supplementary Figure 12. The effect of agarose content on the training process of positive training hydrogels. The hydrogels contained 1 wt%, 2 wt%, or 3 wt% agarose. Error bars represent standard deviations from 4 measurements.

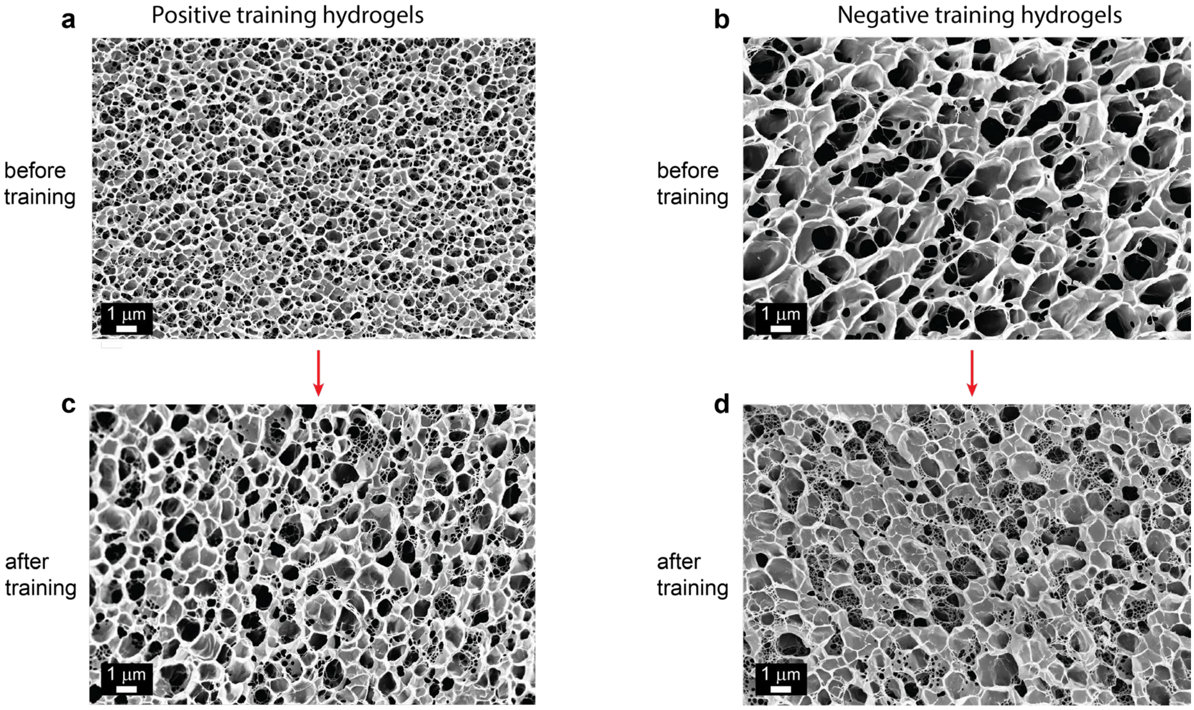

Supplementary Figure 13. SEM photographs of positive and negative training hydrogels at RT. a & c Positive training hydrogels (2 wt% agarose) before and after training. **b & d** Negative training hydrogels (2 wt% ac-agarose, DA 15) before and after training. 3 cycles of training were carried out at 70°C for 5 min.

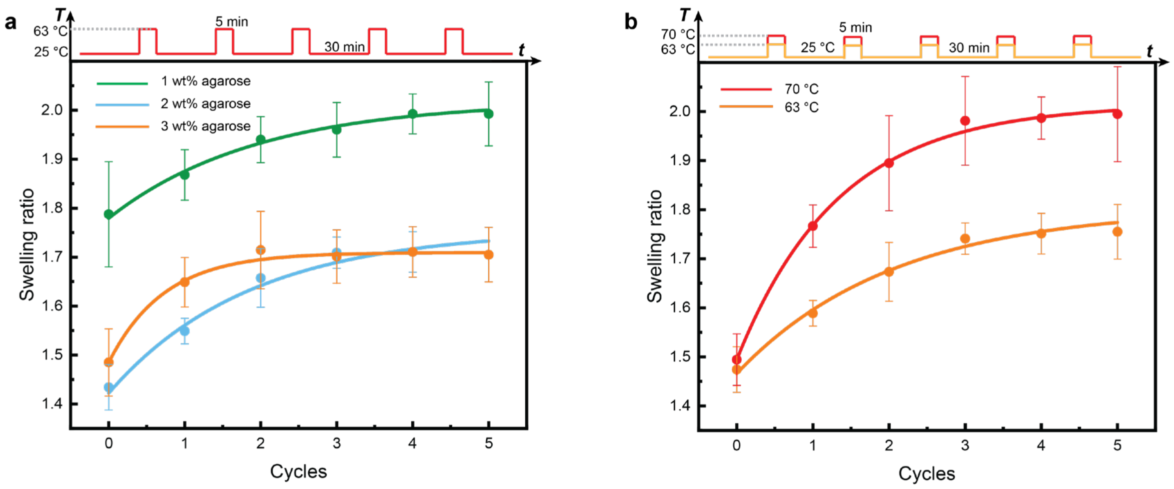

Supplementary Figure 14. Fitting curves of positive training hydrogels during training. a Fitting plots of hydrogels containing 1 wt%, 2 wt%, and 3 wt% agarose. **b** Fitting plots of positive training hydrogels containing 2 wt% agarose at 63 °C and 70 °C. The data points are the swelling ratios at RT.

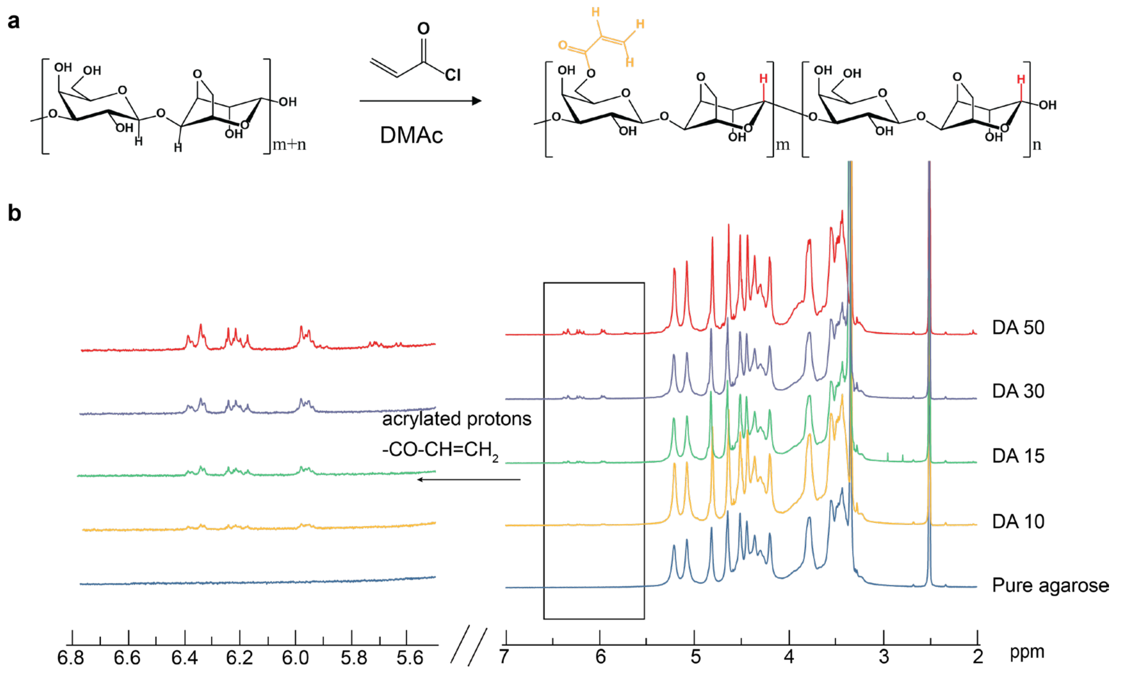

Supplementary Figure 15. Reaction scheme for the synthesis of acrylated agarose and the ^{1}H NMR spectra of different degrees of acrylation. a Acrylation reaction in agarose. **b** Pure agarose and ac-agarose with different DA (10, 15, 30, and 50).

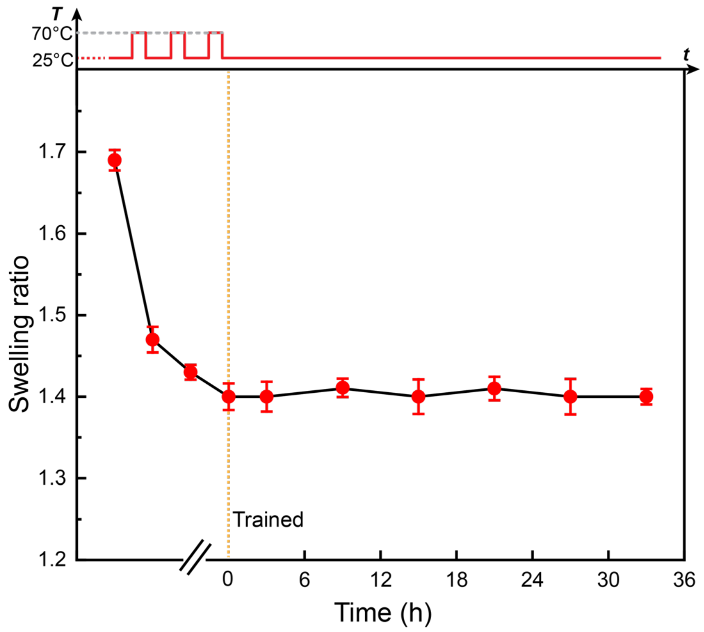

Supplementary Figure 16. The decreased equilibrium swelling ratio after training in the negative training hydrogel remained stable for more than 30 hours. The hydrogel was prepared using 10 wt% PNIPAm and 2 wt% ac-agarose (DA 15). The training process consisted of 3 cycles of 5 min heating at 70°C and 30 min at 25°C. Error bars represent standard deviations from 4 samples.

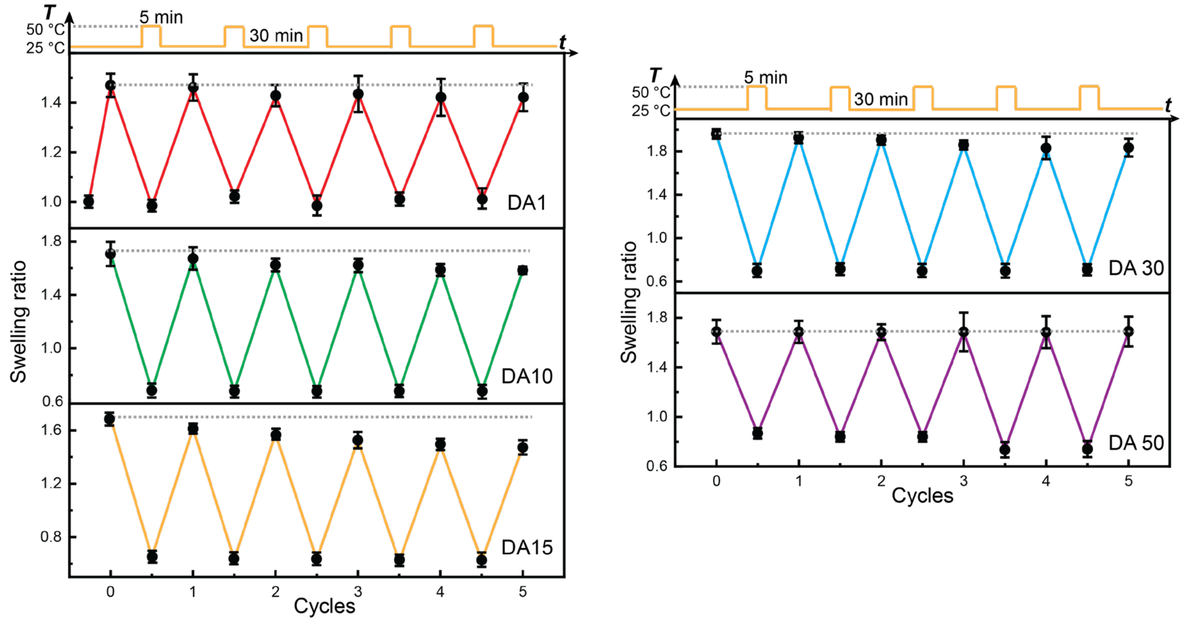

Supplementary Figure 17. Effect of degree of acrylation on negative training. The hydrogels contained five different modifications of ac-agaroses including 1%, 10%, 15%, 30%, and 50%. Error bars are standard deviations from 4 measurements.

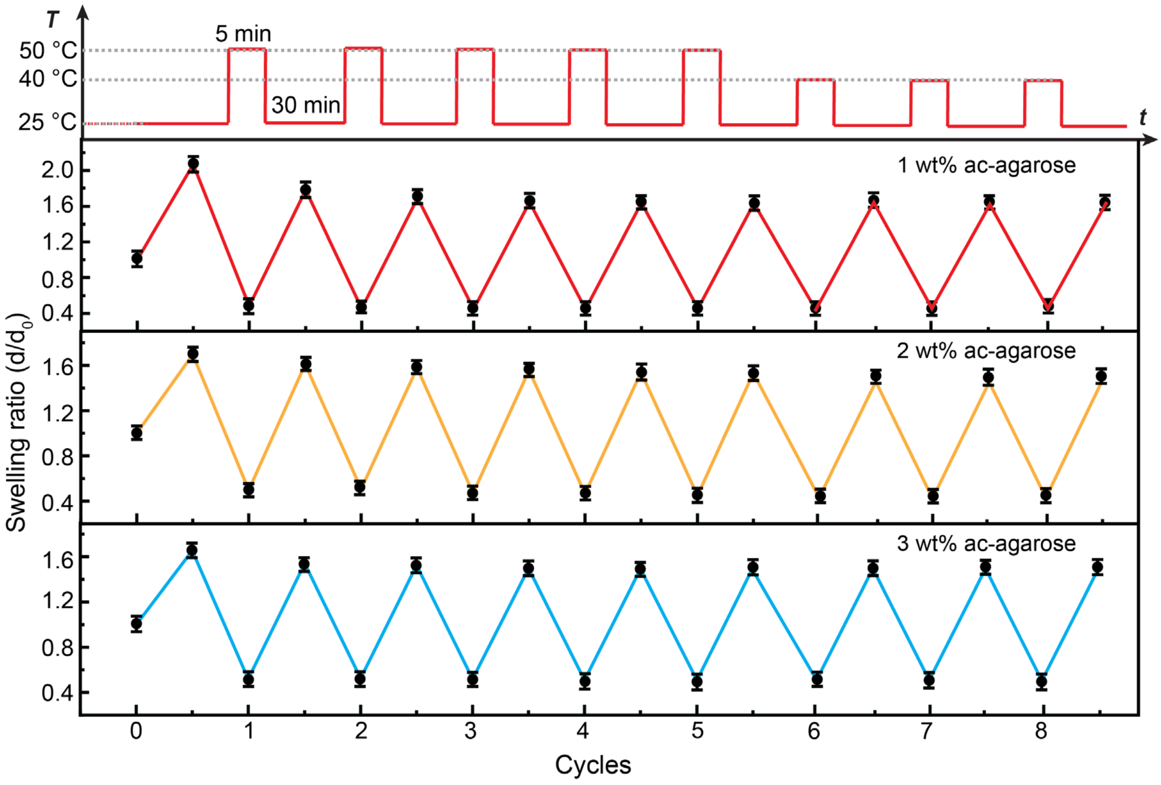

Supplementary Figure 18. The effect of ac-agarose (DA 15) content on swelling ratio during the training process. The IIN hydrogels contained 1 wt%, 2 wt%, and 3 wt% ac-agarose. Error bars represent standard deviations from 4 measurements.

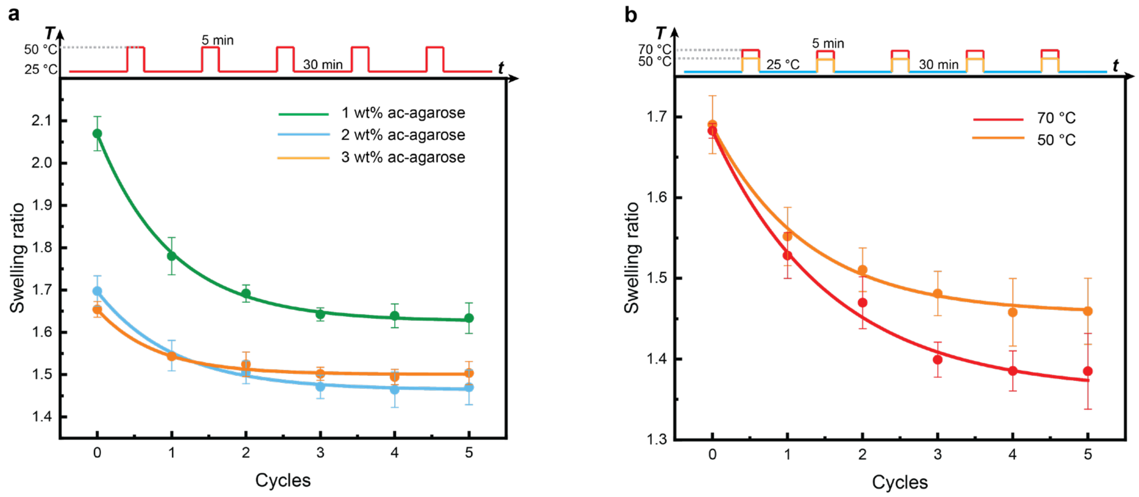

Supplementary Figure 19. Fitting of negative training hydrogels under training. a Negative training hydrogels consist of 1 wt%, 2 wt%, and 3 wt% ac-agarose (DA 15). **b** Training of IIN hydrogels containing 2 wt% ac-agarose (DA 15) at 50 and 70°C for 5 cycles. Error bars are standard deviations from 4 measurements. The data points are the swelling ratios at RT.

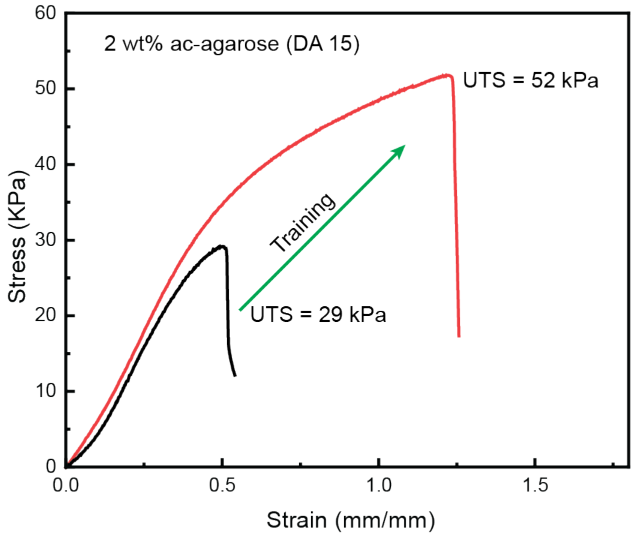

Supplementary Figure 20. Tensile test for negative training hydrogels. Strain-stress curves of IIN hydrogel containing 2 wt% ac-agarose (DA 15), showing an increase in the UTS from 29 kPa before training (black line) to 52 kPa after training (red line).

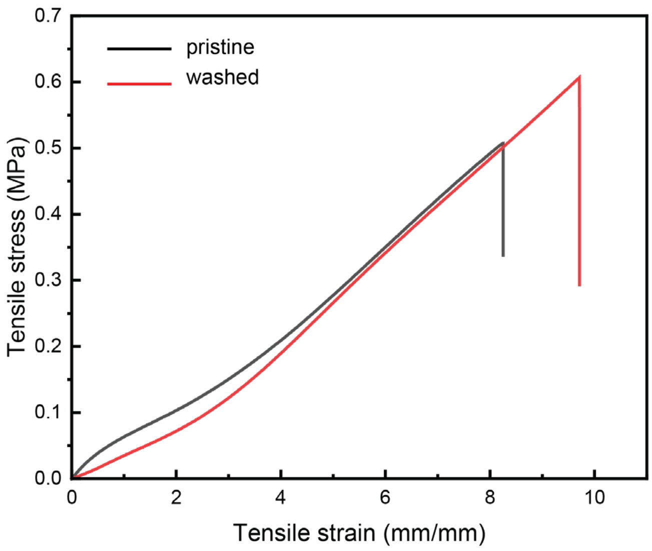

Supplementary Figure 21. **Tensile curve of agarose-PAAm hydrogel after three cycles of thermal training at 70°C.** Hydrogel consists of 2 wt% ac-agarose (DA 15) and 10 wt% polyacrylamide (PAAm).

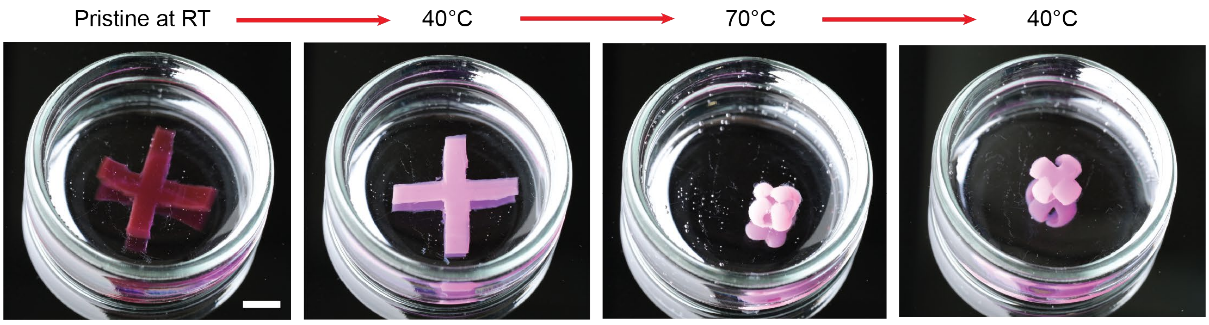

Supplementary Figure 22. The training process of a four-arm bilayer actuator. The hydrogel bilayer consists of a 0.5 mm positive hydrogel layer (2 wt% agarose) and a 0.5 mm negative training hydrogel layer (2 wt% ac-agarose, DA15). Scale bar: 1 cm.

3. Reference

1. Pourjavadi, A., Sadat Afjeh, S., Seidi, F. & Salimi, H. Preparation of acrylated agarose-based hydrogels and investigation of their application as fertilizing systems. *J. Appl. Polym. Sci.* **122**, 2424–2432 (2011).
2. Gamini, A., Toffanin, R., Murano, E. & Rizzo, R. Hydrogen-bonding and conformation of agarose in methyl sulfoxide and aqueous solutions investigated by 1H and 13C NMR spectroscopy. *Carbohydr. Res.* **304**, 293–302 (1997).
