# Supplementary material for: Thermally trainable dual network hydrogels
Source: Nat Commun. 2023 Jun 22;14:3717. doi: 10.1038/s41467-023-39446-w (PMC10287690; doi:10.1038/s41467-023-39446-w)
Supplement: Supplementary file 3 — Description of Additional Supplementary Files [file 41467_2023_39446_MOESM3_ESM.pdf]

Description of Additional Supplementary Files

File Name: Supplementary Movie 1
Description: A hydrogel bilayer gripper trained to grab a weight by training at 70°C.
